# Molecular evolution and functional divergence of CsAlaDC from CsSDC reveal key determinants for theanine biosynthesis in tea plants

**DOI:** 10.1093/hr/uhag153

**Published:** 2026-04-17

**Authors:** Hui Zhou, Peixian Bai, Yongxin Wang, Yueqi Wang, Liubin Wang, Guanhua Liu, Kang Wei, Liyuan Wang

**Affiliations:** National Center for Tea Plant Improvement, National Key Laboratory for Tea Plant Germplasm Innovation and Resource Utilization, Key Laboratory of Biology, Genetics and Breeding of Special Economic Animals and Plants, Ministry of Agriculture and Rural Affairs, Tea Research Institute, Chinese Academy of Agricultural Sciences, Hangzhou 310008, China; Tea Research Institute, Hangzhou Academy of Agricultural Science, Hangzhou 310024, China; National Center for Tea Plant Improvement, National Key Laboratory for Tea Plant Germplasm Innovation and Resource Utilization, Key Laboratory of Biology, Genetics and Breeding of Special Economic Animals and Plants, Ministry of Agriculture and Rural Affairs, Tea Research Institute, Chinese Academy of Agricultural Sciences, Hangzhou 310008, China; National Center for Tea Plant Improvement, National Key Laboratory for Tea Plant Germplasm Innovation and Resource Utilization, Key Laboratory of Biology, Genetics and Breeding of Special Economic Animals and Plants, Ministry of Agriculture and Rural Affairs, Tea Research Institute, Chinese Academy of Agricultural Sciences, Hangzhou 310008, China; National Center for Tea Plant Improvement, National Key Laboratory for Tea Plant Germplasm Innovation and Resource Utilization, Key Laboratory of Biology, Genetics and Breeding of Special Economic Animals and Plants, Ministry of Agriculture and Rural Affairs, Tea Research Institute, Chinese Academy of Agricultural Sciences, Hangzhou 310008, China; National Center for Tea Plant Improvement, National Key Laboratory for Tea Plant Germplasm Innovation and Resource Utilization, Key Laboratory of Biology, Genetics and Breeding of Special Economic Animals and Plants, Ministry of Agriculture and Rural Affairs, Tea Research Institute, Chinese Academy of Agricultural Sciences, Hangzhou 310008, China; National Center for Tea Plant Improvement, National Key Laboratory for Tea Plant Germplasm Innovation and Resource Utilization, Key Laboratory of Biology, Genetics and Breeding of Special Economic Animals and Plants, Ministry of Agriculture and Rural Affairs, Tea Research Institute, Chinese Academy of Agricultural Sciences, Hangzhou 310008, China; National Center for Tea Plant Improvement, National Key Laboratory for Tea Plant Germplasm Innovation and Resource Utilization, Key Laboratory of Biology, Genetics and Breeding of Special Economic Animals and Plants, Ministry of Agriculture and Rural Affairs, Tea Research Institute, Chinese Academy of Agricultural Sciences, Hangzhou 310008, China

## Abstract

Theanine, a non-protein amino acid predominantly found in tea (*Camellia sinensis*), is a primary contributor to the characteristic umami and sweet taste of tea infusion and is associated with numerous health benefits. The biosynthesis of its direct precursor, ethylamine, is catalyzed by the enzyme CsAlaDC. The evolutionary origin of this enzyme and the molecular basis for its functional divergence from the serine decarboxylase CsSDC, however, remain poorly understood. In this study, through comprehensive genome-wide identification, phylogenetic, structural, and domain analyses, we demonstrate that CsAlaDC originated from CsSDC via gene duplication followed by functional specialization. Phe106 and Gly168 were identified as indispensable residues governing CsAlaDC enzymatic activity *in vitro* and *in vivo* through functional validation. Crucially, we pinpointed two specific codon substitutions—TAC(Tyr^112^) to TTT(Phe^106^) and TGT(Cys^174^) to GGT(Gly^168^)—as key evolutionary mutations responsible for the functional shift from SDC to AlaDC activity. These findings elucidate the evolutionary trajectory of CsAlaDC and provide mechanistic insights into the molecular regulation of theanine biosynthesis in tea plants.

## Introduction

Tea, a globally important economic crop, contain numerous bioactive compounds that determine its quality and health benefits, including catechins, caffeine, and theanine. Theanine represents the most abundant free amino acid in tea plants and is present in various tissues [1]. Beyond *Camellia sinensis*, theanine has also been identified in other *Camellia* species and in select edible fungi such as *Xerocomus badius* [2–5]. Theanine provides a range of health benefits, such as neuroprotection, memory and cognitive enhancement, antidepressant effects, blood pressure regulation, as well as antitumor, antimicrobial, and immunomodulatory activities [6, 7]. As a distinctive metabolite characteristic of tea, theanine is mainly biosynthesized in the roots [8, 9]. Its production requires two substrates: ethylamine and glutamate. Glutamate is widely available in most plants, whereas ethylamine predominantly accumulates in species of the genus *Camellia*, especially in *C. sinensis*.

Experimental evidence indicates that several plant species—including *Camellia nitidissima*, *C. japonica*, *Zea mays*, *Arabidopsis thaliana*, and *Solanum lycopersicum*—possess enzymatic machinery capable of catalyzing theanine formation from ethylamine and glutamate. This was confirmed by feeding experiments with [^2^H_5_] ethylamine, which consistently yielded [^2^H_5_] theanine in these plants. These results suggest that the limited availability of ethylamine, rather than the absence of the biosynthetic pathway itself, is a key factor restricting theanine accumulation outside *C. sinensis* [3]. One pathway for ethylamine synthesis is the decarboxylation of alanine, catalyzed by alanine decarboxylase (AlaDC). Initial characterization of this enzyme activity came from crude extracts of *Cucumis sativus* (cucumber) and *Ecballium elaterium* (squirting cucumber), where the addition of 10 μg/ml pyridoxal-5′-phosphate (PLP)—an essential cofactor for decarboxylases—increased activity by 1.8-fold [10]. While AlaDC displays low catalytic efficiency toward serine, its primary substrate is alanine [11]. Critical evidence for its role in tea emerged from isotopic tracing experiments by Takeo in the 1970s, which demonstrated that the ethylamine moiety of theanine is derived predominantly from alanine decarboxylation [12]. Subsequent work later quantified AlaDC activity in tea seedlings [13].

In our previous work, we isolated and functionally characterized *CsAlaDC*, a gene from tea plant that encodes a protein with confirmed alanine decarboxylase activity [14]. Sequence alignment showed high homology between CsAlaDC and plant serine decarboxylases (SDCs), yet functional assays revealed a strict substrate specificity of CsAlaDC toward alanine. Evolutionary analysis further indicated that *CsAlaDC* likely arose from gene duplication of the ancestral *CsSDC* and that its activity is significantly enhanced by PLP. Notably, compared to CsSDC, CsAlaDC exhibits lower specific activity and reduced thermostability [15]. In plant metabolism, it is widely recognized that specialized metabolic pathways frequently evolve from pre-existing primary pathways [16, 17].The catalytic enzymes involved in these derived pathways are commonly recruited from ancestral protein families through genetic mechanisms such as gene duplication and subsequent neofunctionalization, which serve as major drivers for the emergence of novel enzymatic functions in plants [16, 18].

The evolutionary pathway linking *CsAlaDC* to its ancestral homolog *CsSDC* in tea plants has not been fully resolved. Although prior work has established the serine decarboxylase activity of CsSDC and the alanine decarboxylase activity of CsAlaDC, the precise evolutionary relationship between these two enzymes—as well as the key residues that determine their distinct catalytic specificities—remains unclear. To address these questions, we combined genomic, phylogenetic, and structural analyses with *in vitro* and in planta functional assays to trace the evolutionary origin of *CsAlaDC* and to pinpoint the residues responsible for its functional divergence from *CsSDC*. Our findings offer new perspectives on the evolution of theanine metabolism and provide a molecular framework for the potential biotechnological production of ethylamine and theanine.

## Results

### Functional characterization of CsAlaDC in *A. thaliana*

**Figure 1:**
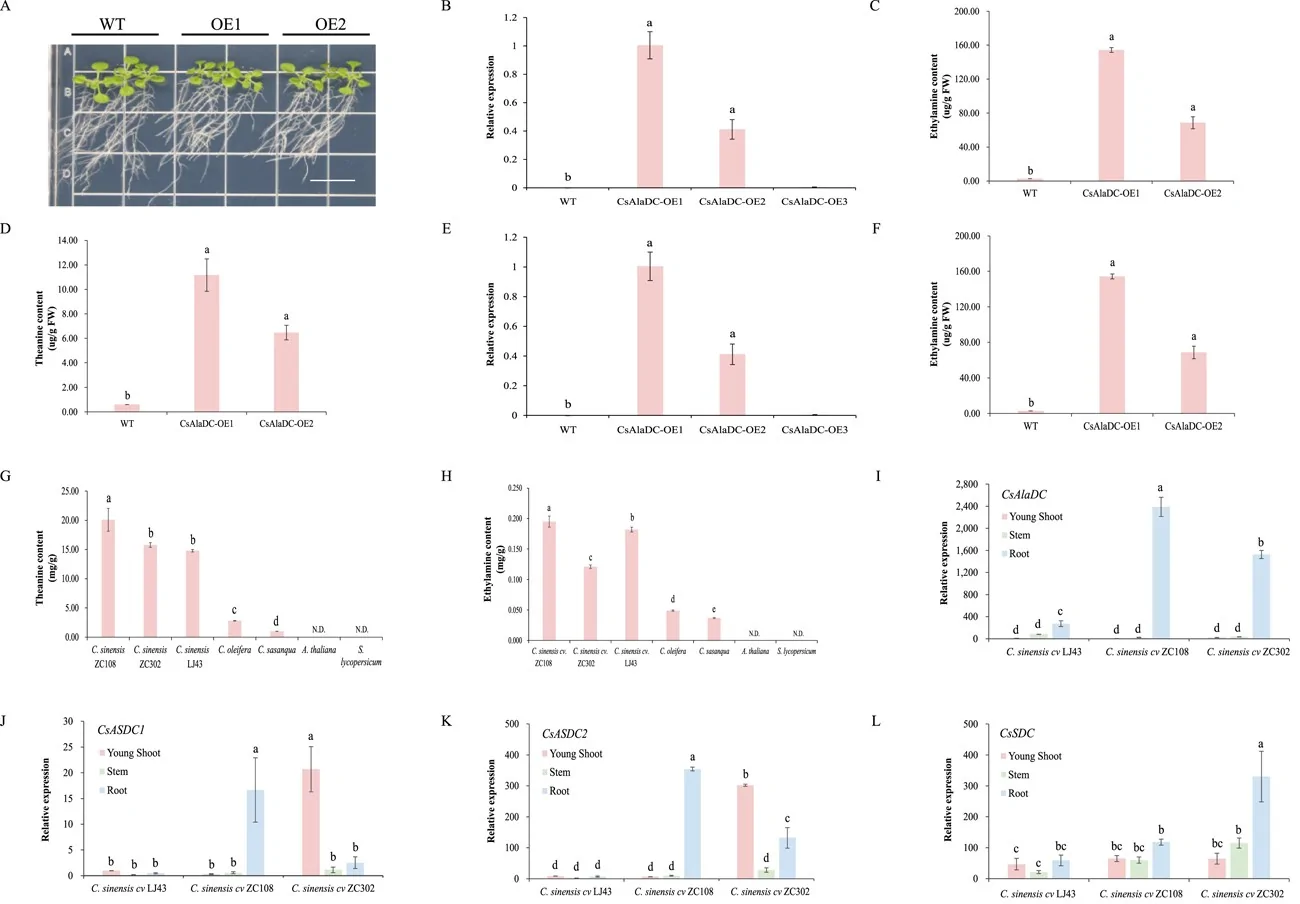
Functional analysis of *CsAlaDC/SDC*. (A) Phenotypic comparison between WT and *CsAlaDC*-overexpressing (OE) seedlings. Scale bar = 1 cm. (B) Relative transcript levels of *CsAlaDC* in WT and OE lines, determined by qRT–PCR. (C-D) Ethylamine and theanine contents in WT and OE seedlings. (E, F) Fresh weight and primary root length of OE and WT seedlings. (G, H) Theanine and ethylamine contents in various species: *Camellia sinensis* cv. ‘Longjing 43’ (LJ43), *Camellia sinensis* cv. ‘Zhongcha 108’ (ZC108), *Camellia sinensis* cv. ‘Zhongcha 302’ (ZC302), and *C. oleifera*, *C. sasanqua*, *A. thaliana*, and *S. lycopersicum*. (I–L) Relative expression levels of *CsAlaDC/SDC* genes in young shoots, stems, and roots of the three tea cultivars. Data are presented as mean ± SD (*n* = 3). Significant differences (*P* < 0.05) were determined by r one-way ANOVA with Duncan’s multiple range test, different lowercase letters denote statistically significant differences. ‘ND’ indicates not detected.

Subcellular localization analysis revealed that the CsAlaDC protein is localized to both the cytoplasm and the plasma membrane (Fig. S1). This dual localization implies that the alanine decarboxylation reaction producing ethylamine likely takes place within these cellular compartments in tea plants. To investigate the *in vivo* function of *CsAlaDC*, we generated transgenic *A. thaliana* overexpressing this gene (CsAlaDC-OE) and analyzed transgene expression along with metabolite accumulation. Reverse transcription quantitative polymerase chain reaction (RT-qPCR) confirmed robust expression of CsAlaDC, and consequently, CsAlaDC-OE lines accumulated significantly higher levels of ethylamine and theanine compared to the wild-type (WT) controls (Fig. 1B–D). As anticipated, ethylamine was not detectable in WT plants. Phenotypic evaluation of 2-week-old seedlings showed that overexpression of *CsAlaDC* strongly inhibited the growth of *A. thaliana* (Fig. 1A). Relative to WT, the transgenic lines displayed a 24.7%–38.1% reduction in fresh weight and a 10.4%–14.3% decrease in primary root length (Fig. 1E, F). These finding demonstrate that CsAlaDC negatively regulates plant growth and development, potentially by disrupting amino acid homeostasis.

### Distribution patterns of theanine and its precursor ethylamine

Theanine accumulation was highest in tea plants, with root concentrations ranging from 14.8 to 20.1 mg g^−1^ DW. In comparison, roots of *C. oleifera* and *C. sasanqua* markedly lower levels (1.0–2.8 mg g^−1^ DW), while theanine was not detectable in *A. thaliana* and *S. lycopersicum* (Fig. 1G). Ethylamine, the direct biosynthetic precursor of theanine, showed a distribution profile highly consistent with that of theanine across species. Statistical analysis revealed a strong positive correlation between ethylamine and theanine contents (Fig. 1H; Table S5).

### Expression profiles and physicochemical characteristics of *CsAlaDC/SDC*

Expression analysis confirmed the pivotal role of CsAlaDC in theanine biosynthesis, with transcript levels substantially higher in roots compared to other tissues across all tested tea cultivars. *CsSDC* exhibited a similar root-predominant expression pattern (Fig. 1I, J). Homology searches using BLAST against multiple tea plant genomes (‘Yunkang 10,’ ‘Shuchazao,’ and ‘Longjing 43’) identified two additional genes with intermediate sequence similarity to *CsAlaDC* and *CsSDC*, which were designated *CsASDC1* and *CsASDC2*. The expression profiles of *CsASDC1* and *CsASDC2* did not display a consistent tissue-specific pattern among the cultivars (Fig. 1K, L). Key physicochemical properties of the deduced proteins are summarized in Table S6. Multiple sequence alignment revealed that all four proteins contain a conserved pyridoxal phosphate (PLP)-dependent decarboxylase domain (Fig. S2). Consistent with this, predicted 3D structural models indicated a highly similar overall fold for each protein, with all adopting a homodimeric conformation (Fig. S3).

### Chromosome distribution and phylogenetic analysis of *CsAlaDC/SDC*

**Figure 2:**
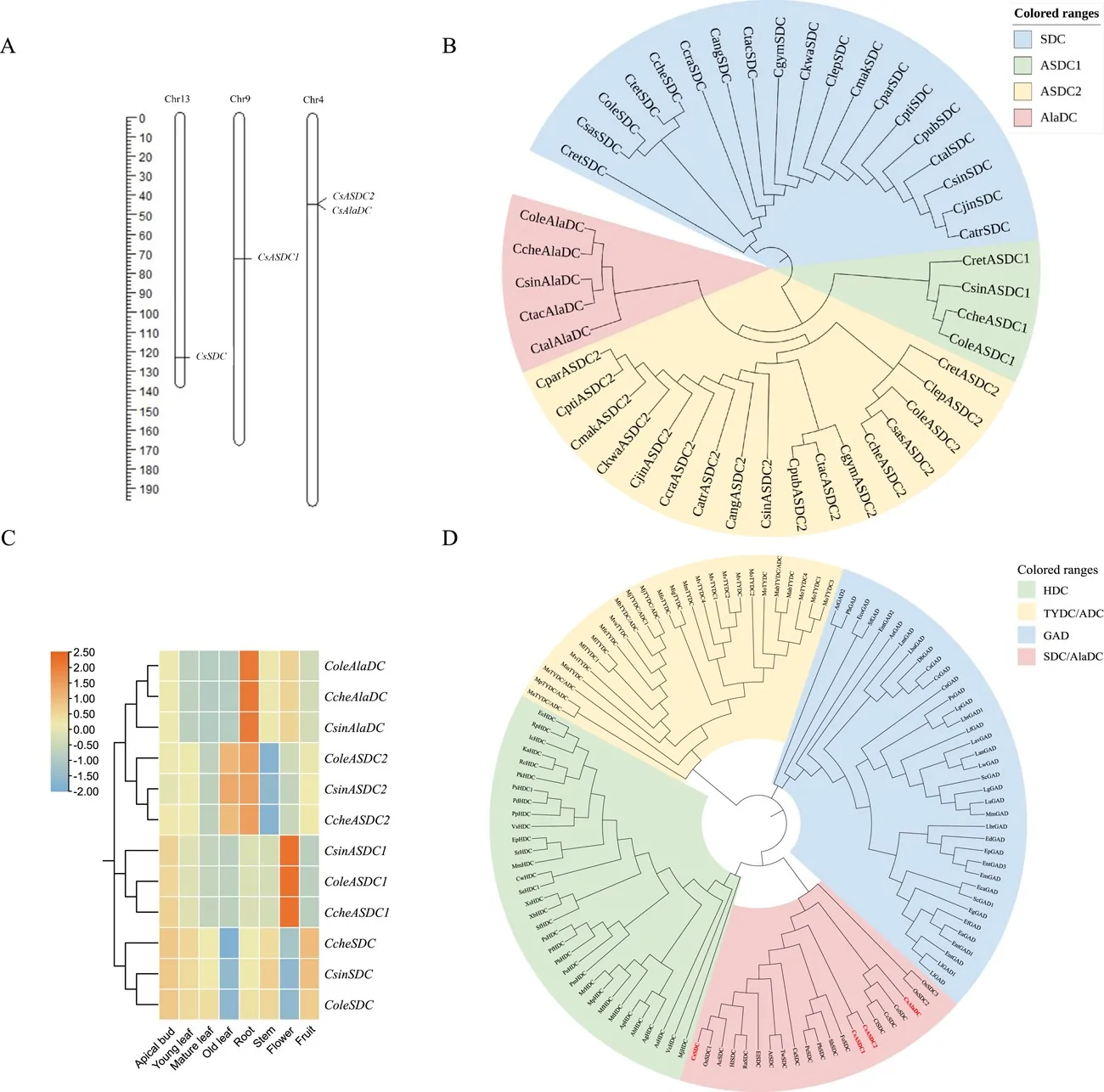
Genetic analysis of *AlaDC/SDC* gene family in *Camellia* species. (A) Chromosomal positions of *CsSDC*, *CsASDC1*, *CsASDC2*, and *CsAlaDC* in the *C. sinensis* genome. (B) Distribution of *AlaDC/SDC* homologs across 19 *Camellia* species. Color coding: blue, *CsSDC*; green, *CsASDC1*; yellow, *CsASDC2*; pink, *CsAlaDC*. (C) Expression profiles of *AlaDC/SDC* genes in *C. sinensis*, *C. oleifera*, and *C. chekiangoleosa*, visualized using a heatmap. (D) Phylogenetic tree of AlaDC/SDC proteins. Proteins from *C. sinensis* are highlighted in red. Other homologous proteins are grouped by function: histidine decarboxylase (HDC, green), tyrosine/aspartate decarboxylase (TDC/ADC, yellow), glutamate decarboxylase (GAD, blue), and serine decarboxylase (SDC, pink).

Within the genome of *Camellia sinensis* cv. ‘Shuchazao,’ members of the *CsAlaDC/SDC* gene family are distributed across several chromosomes. *CsSDC* is located on chromosome 13 at position 122.7 Mb, while *CsSDC1* resides on chromosome 9 at 72.3 Mb. Notably, *CsSDC2* and *CsAlaDC* are positioned adjacently on chromosome 4 at 44.1 Mb and 44.2 Mb, respectively (Fig. 2A). Phylogenetic reconstruction revealed distinct evolutionary relationships. *CsAlaDC*, *CsASDC1*, and *CsASDC2* clustered within a clade containing *OsSDC2* and *OsSDC3*. This grouping suggests that CsASDC1 and CsASDC2 likely encode serine decarboxylases with catalytic functions analogous to their rice orthologs [19]. Furthermore, CsAlaDC itself formed a distinct subclade specifically with sequences from other *Camellia* species (*C. chekiangoleosa*, *C. oleifera*, and *C. lanceisepala*), indicating a closer evolutionary relationship within the *Camellia* genus compared to other plant lineages (Fig. 2D).

### Expression of *AlaDC/SDC* homologs across *camellia* species

Homologs of *CsAlaDC/SDC* were identified by analyzing transcriptomic data from 19 *Camellia* species. The *SDC* gene was ubiquitously expressed across all species examined. In contrast, *ASDC2* transcripts were detected in 17 species, while the expression of *ASDC1* and *AlaDC* was restricted to *C. sinensis*, *C. oleifera*, and *C. chekiangoleosa* (Fig. 2B). Further analysis of RNA-seq data from the Tea Plant Information Archive (TPIA) database revealed tissue-specific expression patterns of *AlaDC/SDC* genes in *C. sinensis*, *C. oleifera*, and *C. chekiangoleosa*. In all three species, AlaDC homologs (*CsAlaDC*, *CoAlaDC*, and *CcAlaDC*) were predominantly expressed in root tissues (Fig. 2C). This root-predominant expression pattern aligns with the biological function of these genes in ethylamine synthesis, a key precursor for theanine production in tea plant roots.

### Evolutionary dynamics of *AlaDC/SDC* genes revealed by genome-wide identification

**Figure 3:**
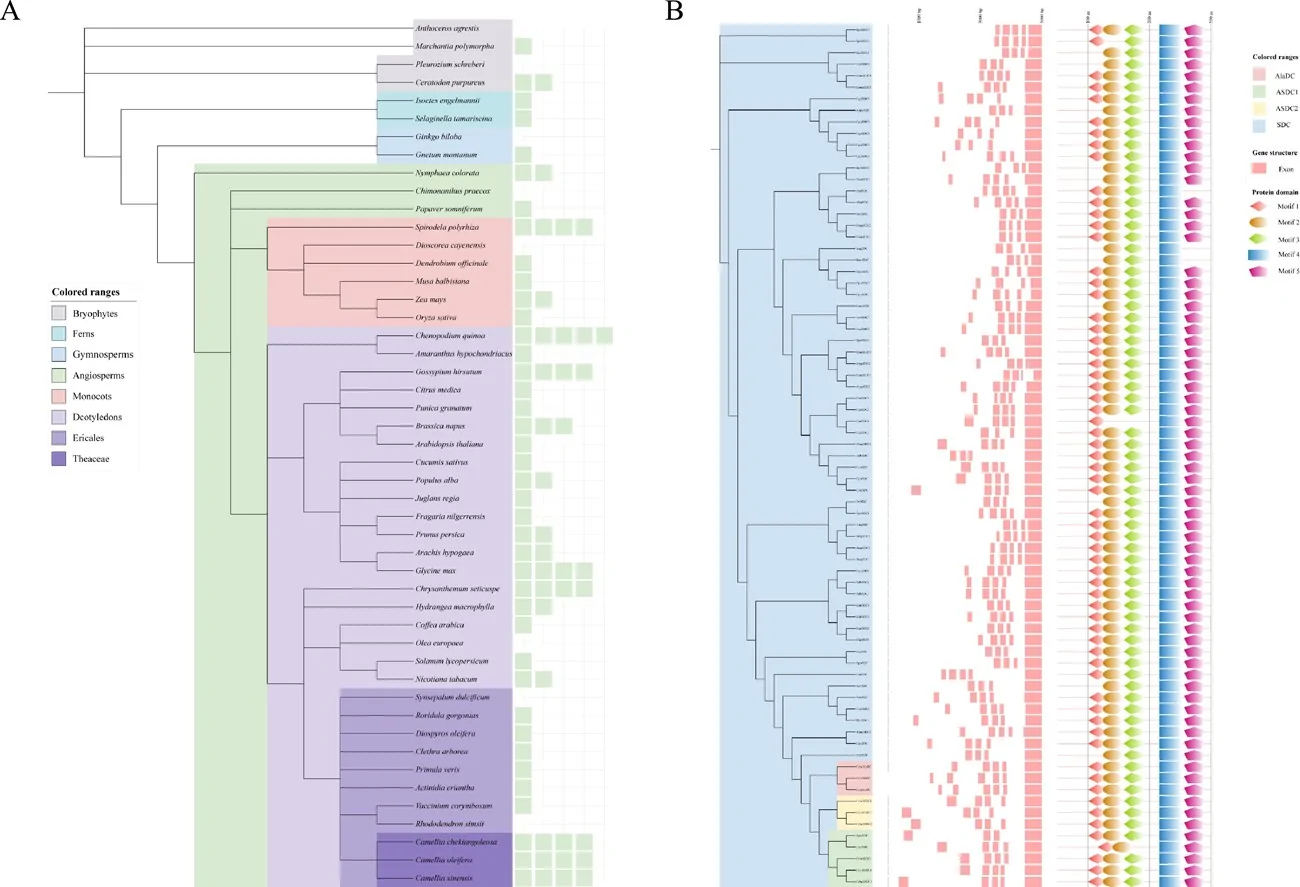
Structural evolution analysis of *AlaDC/SDC* gene family across plant species. (A) Lineage-specific expansion of the AlaDC/SDC gene family. A genome-wide search across 48 species spanning seven major taxonomic groups (color-coded bars) identified AlaDC/SDC homologs in 40 species. The number of adjacent green boxes indicates the gene copy number per species (one box = one copy; blank spaces denote absence). (B) Phylogenetic clustering and structural conservation of AlaDC/SDC proteins. The neighbor-joining phylogenetic tree (1000 bootstrap replicates) integrates conserved motif architectures (colored blocks) and exon-intron structures (pink: exons; white lines: introns; scaled by sequence length).

A genome-wide search for *CsAlaDC/SDC* homologs across 48 plant species revealed their presence in 40 species. Among these, 23 species harbored a single-copy gene, 17 species possessed multiple copies, and eight species lacked detectable homologs. Within the order Ericales (which includes Theaceae), six of the eight examined non-Theaceae species (e.g. *Roridula gorgonias* and *Diospyros oleifera*) retained a single-copy homolog. In contrast, no homologs were found in *Synsepalum dulcificum* or *Rhododendron simsii*. All three analyzed *Camellia* species (*C. sinensis*, *C. oleifera*, and *C. chekiangoleosa*) consistently contained four conserved *AlaDC/SDC* copies (Fig. 3. A). This conservation suggests a lineage-specific gene duplication event occurred in the common ancestor of Theaceae after its divergence from other Ericales lineages but prior to the radiation of extant *Camellia* species. Such an expansion likely provided the genetic substrate for the functional diversification of *AlaDC/SDC* genes within tea plants.

### Phylogenetic analysis of AlaDC/SDC proteins

Analysis of exon-intron structures across species indicated high conservation among *CsAlaDC/SDC* genes, with most encoded proteins sharing five common motifs (Fig. 3B). In species containing multiple *AlaDC/SDC* copies, the corresponding proteins consistently clustered into distinct phylogenetic subclades. This pattern is evident in *Spirodela polyrhiza* (three copies), *Chenopodium quinoa,* and *Gossypium hirsutum* (four copies each), where paralogs formed separate, well-defined branches. Within *Camellia* (*C. sinensis*, *C. oleifera*, and *C. chekiangoleosa*), the four conserved paralogs corresponded to four clearly differentiated protein groups: SDC, ASDC1, ASDC2, and AlaDC. *Camellia* SDCs clustered with SDCs from other plant lineages, while ASDC1, ASDC2, and AlaDC from *Camellia* together formed a robust, genus-specific clade. Interestingly, SDC proteins from *Roridula gorgonias* (*Roridulaceae*) and *Clethra arborea* (*Clethraceae*) grouped closely with ASDC1 from *Camellia*, suggesting these proteins may be direct homologs of ASDC1. Together, these phylogenetic relationships imply that CsASDC1 likely arose earlier than CsASDC2 and CsAlaDC during the evolutionary diversification of this gene family.

### Identification of key residues underlying functional divergence in CsAlaDC/SDC enzymes

**Figure 4:**
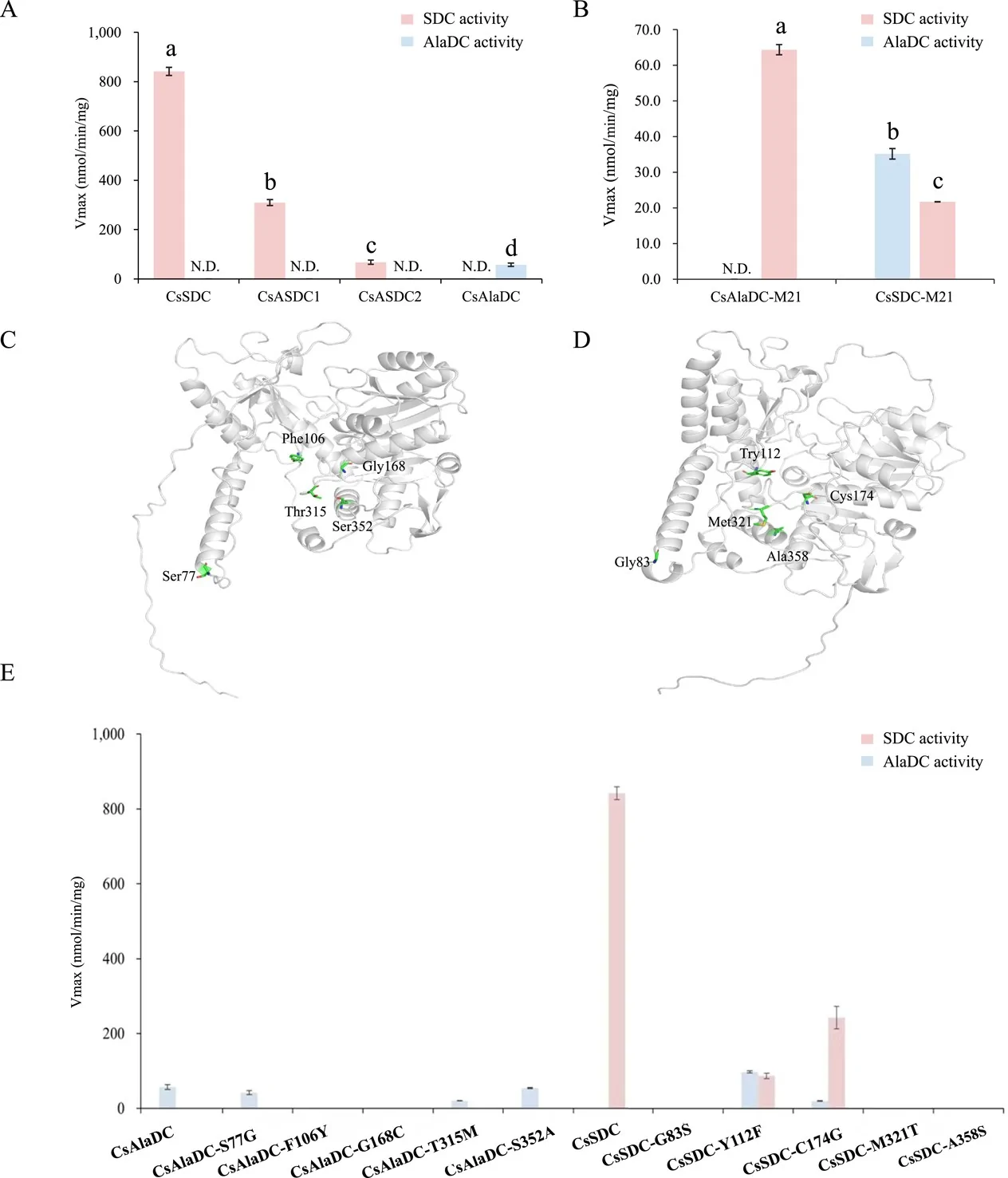
Identification of key residues governing CsAlaDC/SDC enzymatic activity and functional divergence. (A) Enzymatic activity of recombinant CsSDC, CsASDC1, CsASDC2, and CsAlaDC proteins. (B) Substrate specificity of engineered mutant enzymes CsAlaDC-M21 and CsSDC-M21. (C, D) Structural mapping of divergent residues between CsAlaDC and CsSDC onto their predicted protein models. In CsSDC, residues Tyr112, Cys174, and Met321 are located near the catalytic pocket, with Gly83 and Ala358 positioned peripherally. In CsAlaDC, the corresponding sites are Phe106, Cys174, Thr315 (catalytic region), and Ser77 and Ser352 (peripheral regions). These residues are proposed to underlie functional divergence. (E) Enzymatic validation of mutant proteins generated through reciprocal substitution of the key divergent residues. Data are presented as mean ± SD (*n* = 3). Different lowercase letters indicate statistically significant differences (*P* < 0.05; one-way ANOVA followed by Duncan’s multiple range test). ‘ND’ denotes not detected.

Enzyme activity assays of the four recombinant proteins confirmed that CsASDC1 and CsASDC2 possess serine decarboxylase activity similar to CsSDC (Fig. 4A). Alignment of CsSDC and CsAlaDC proteins from diverse species identified 21 positions that are conserved in CsSDC but consistently substituted in CsAlaDC (Fig. S4), pointing to their potential role in functional divergence. To test this hypothesis, we engineered reciprocal mutations at these 21 sites, generating CsAlaDC-M21 and CsSDC-M21. The purified mutant proteins were assayed for substrate specificity. Strikingly, the mutations resulted in a near-complete functional swap (Fig. 4B). CsAlaDC-M21 lost its native alanine decarboxylase activity and instead gained serine decarboxylase function, while CsSDC-M21 acquired strong alanine decarboxylase activity, retaining less than 1% of its original serine decarboxylase activity. These findings establish that these 21 residues are the primary determinants of the functional specialization between CsAlaDC and CsSDC.

### Screening of candidate functional residues in CsAlaDC/SDC

Catalytic efficiency and substrate selectivity in decarboxylases are primarily determined by active site architecture and the geometry of the substrate-binding pocket. To identify residues responsible for functional divergence between CsAlaDC and CsSDC, we mapped the 21 divergent amino acid positions onto the 3D structure of CsSDC, which shares high overall similarity with CsAlaDC. Using the conserved PLP-binding motifs, the substrate-binding residue His^202^, and the catalytic residue Lys^315^ as references, we defined the catalytic core region. Within this region, three residues were located in close proximity to the catalytic center, suggesting their potential role in substrate orientation or catalysis. These were selected as primary candidates for experimental validation. Comparative structural analysis further identified [20]. By analogy, these two sites were also considered potential determinants of substrate accommodation (Fig. 4C-D; Table S7).

### Validation of critical residues for CsAlaDC/SDC enzymatic function

To evaluate the functional contribution of the five candidate residues, we conducted reciprocal single-point mutations in CsAlaDC and CsSDC. The mutant enzymes exhibited clear functional differences depending on the specific residue altered. In CsSDC, mutations G83S, M321T, and A358S completely eliminated serine decarboxylase activity, confirming that these residues are essential for its catalytic function. The corresponding substitutions in CsAlaDC (S77G, T315M, S352A), however, retained 73.7–95.1% of WT activity, indicating a more modest role in alanine decarboxylation. Conversely, residues F^106^ and G^168^ were crucial for CsAlaDC activity, as mutants F106Y and G168C lost all detectable function. Interestingly, the equivalent mutations in CsSDC (Y112F, C174G) retained only 10.4–28.9% of original SDC activity but acquired substantial de novo alanine decarboxylase activity, reaching levels equivalent to 171% and 35.6% of WT CsAlaDC, respectively. This identifies residues 112 and 174 as primary determinants enabling the functional shift from SDC to AlaDC specificity. Furthermore, the underlying codon transitions—TAC (Tyr^112^) → TTT (Phe^106)^ and TGT (Cys^174^) → GGT (Gly^168^)—represent key molecular changes likely responsible for the neofunctionalization of AlaDC (Fig. 4E).

### *In vivo* validation of key residues underlying the neofunctionalization of CsSDC to CsAlaDC

**Figure 5:**
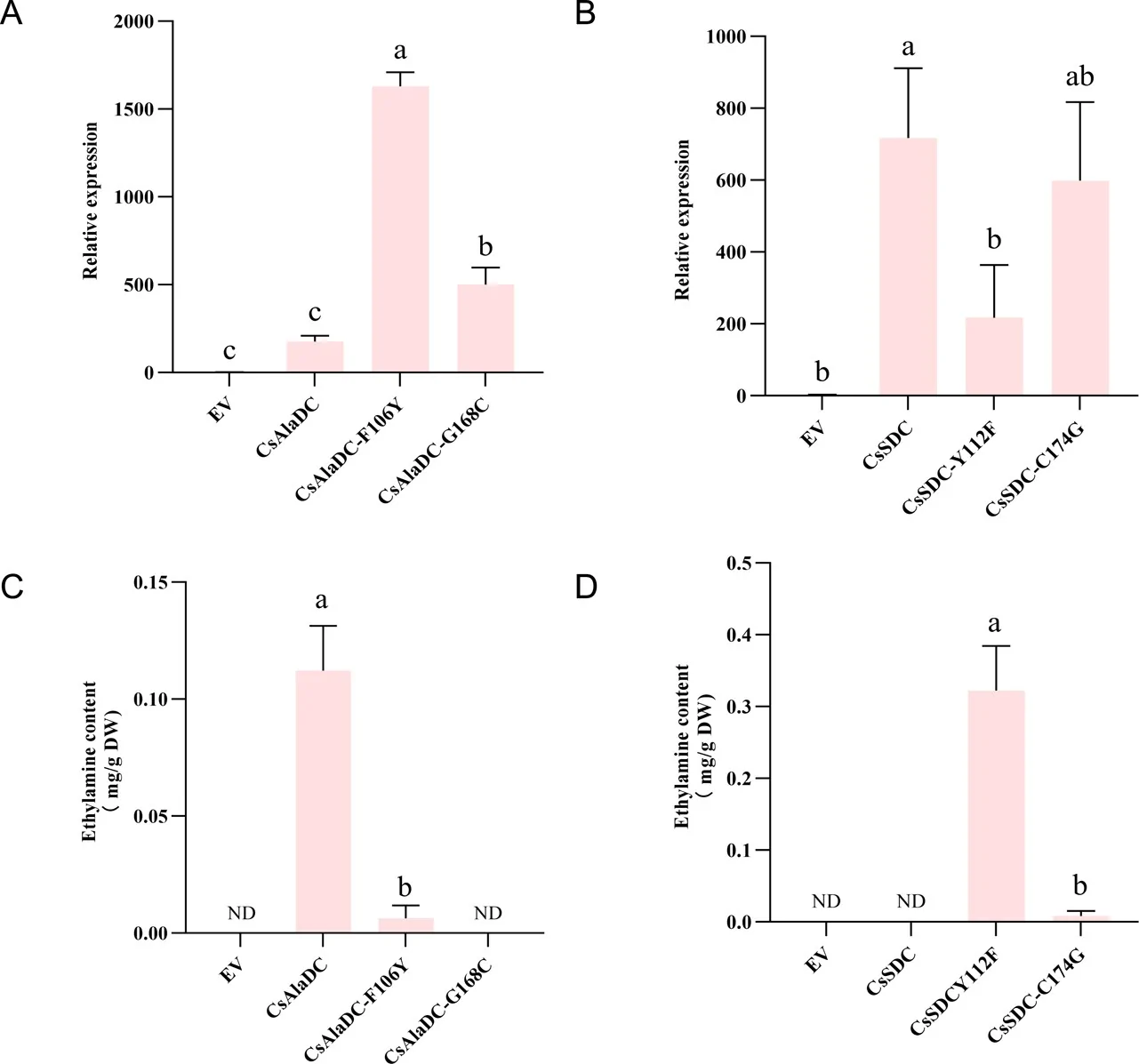
Transgenic analysis of CsAlaDC in *Nicotiana benthamiana*. (A) Relative mRNA levels of CsAlaDC, and its mutant CsAlaDC-F106Y and CsAlaDC-G168C. (B) Relative mRNA levels of CsSDC, and its mutant CsSDC-Y112F and CsSDC-C174G. The EV was used as a control; *NbGAPDH* was used as an internal reference gene. (C) The ethylamine contents of CsAlaDC, and its mutant CsAlaDC-F106Y and CsAlaDC-G168C in *N. benthamiana*. (D) The ethylamine contents of CsSDC, and its mutant CsSDC-Y112F and CsSDC-C174G and in *N. benthamiana*. Different letters above bars indicate statistically significant differences at *P* < 0.05 based on one-way ANOVA followed by Duncan’s multiple range test. ‘ND’ denotes not detected. Error bars represent SD from three independently replicates (*n* = 3).

To further examine the functional relevance of these residues in planta, CsAlaDC, CsSDC, and their mutant variants were transiently expressed in *N. benthamiana*. Quantitative PCR analysis showed that all constructs were strongly expressed, with transcript levels much higher than those of the empty vector (EV) control (Fig. 5A-B). The levels of ethylamine in the tobacco matched *in vitro* enzymatic activities. OE-*CsAlaDC* caused high levels of ethylamine, but the CsAlaDC-G168C mutant showed no ethylamine and the CsAlaDC-F106Y produced only very small amounts. However, OE-*CsSDC* did not produce ethylamine, while the CsSDC-Y112F mutant produced a lot of ethylamine and CsSDC-C174G produced low but clearly detectable levels. There was no ethylamine in the EV controls (Fig. 5C, D). These results demonstrate that residues Y112F and C174G are critical for the functional divergence between CsSDC and CsAlaDC. The stronger effect of the Tyr-to-Phe substitution suggests that this residue represents a key evolutionary switch conferring CsAlaDC activity in tea plants.

## Discussion

### CsAlaDC is a key enzyme in theanine biosynthesis

Theanine, a signature metabolite of tea (*Camellia sinensis*), is primarily responsible for the characteristic umami taste of tea infusions and possesses multiple bioactive properties. Our results establish that CsAlaDC catalyzes a critical step in its biosynthesis: the decarboxylation of alanine to produce ethylamine, the direct precursor for theanine formation. This functional role aligns with and extends the earlier report by Wang *et al.* [21], which identified CsAlaDC activity as essential for ethylamine production in tea leaves.

In our previous study, CsAlaDC was confirmed to possess alanine decarboxylase activity *in vitro* [14]. Here, to validate its function *in planta*, we overexpressed *CsAlaDC* in *A. thaliana*, resulting in significantly elevated levels of both ethylamine and theanine (Fig. 1B–D). These findings align with the work of Wang *et al*. [21], who observed enhanced ethylamine accumulation upon *CsAlaDC* overexpression in tobacco. Further supporting the central role of ethylamine, exogenous supplementation of this metabolite has been shown to promote theanine accumulation across diverse plant species [3], underscoring its conserved function as a key precursor in theanine biosynthesis. Nevertheless, functional outcomes appear to differ across experimental systems. While *CsAlaDC* overexpression alone in tobacco was sufficient to elevate ethylamine, it did not result in detectable theanine accumulation; theanine synthesis required the additional expression of *CsTS1* [22]. In contrast, *CsAlaDC* overexpression in *A. thaliana* in the present study successfully enhanced both metabolites. However, these transgenic *A. thaliana* plants exhibited significant growth alterations, including reduced fresh weight and shorter primary roots compared to the wild type (Fig. 1A, E, F). Previous research has identified CsLBD37, a nitrogen-responsive transcription factor, as a negative regulator of theanine biosynthesis that also suppresses lateral root development in young plants [23]. Chen *et al.* [24] subsequently clarified that this inhibitory effect is not caused by intracellular theanine overaccumulation. Rather, under high nitrogen conditions, theanine is secreted into the apoplast where it acts as a signaling molecule to fine-tune lateral root formation.

Recent efforts to boost theanine production have focused on manipulating *CsAlaDC* expression. A notable success was achieved by co-overexpressing *CsAlaDC* and theanine synthase (*CsTS*) in rice endosperm, yielding a transgenic line (‘theaRice’) with markedly elevated theanine content. Recent efforts to boost theanine production have focused on manipulating *CsAlaDC* expression. A notable success was achieved by co-overexpressing *CsAlaDC* and theanine synthase (*CsTS*) in rice endosperm, yielding a transgenic line (‘theaRice’) with markedly elevated theanine content [25]. This and related evidence solidify CsAlaDC as a major control point in the theanine pathway and indicate that engineering its activity offers a viable route for increasing theanine accumulation in heterologous systems.

### Evolutionary analysis of *AlaDC/SDC* genes

CsAlaDC is essential for theanine biosynthesis in tea plants; however, its evolutionary origin has been unresolved. Our study clarifies this evolutionary process by demonstrating that the tea-specific *CsAlaDC* likely arose from an ancestral serine decarboxylase gene (*SDC*) through gene duplication followed by neofunctionalization. This pattern is consistent with the classical duplication-divergence model, wherein lineage-specific metabolic innovations often emerge through the recruitment and functional diversification of enzymes from pre-existing pathways [16, 26]. Our findings provide empirical support for this model, indicating that CsAlaDC was evolutionarily recruited from the more ancient phosphatidylethanolamine pathway where CsSDC operates. A central discovery of this study is the identification of two intermediate genes, *CsASDC1* and *CsASDC2*, which serve as evolutionary intermediates bridging the ancestral *CsSDC* and the specialized *CsAlaDC*. This stepwise transition provides strong evidence for a gradual functional divergence process.

Analysis of gene structural and sequence indicates that the functional divergence between *CsAlaDC* and *CsSDC* arose from variations within highly homologous sequences, most likely initiated by substitutions in the first exon. Based on these findings, we propose that *CsAlaDC* evolved from *CsSDC* through at least three sequential rounds of gene duplication followed by functional divergence. While gene duplication is a well-established driver of metabolic innovation in plants [26, 27], and the recruitment of homologous enzymes into distinct biosynthetic pathways is commonly observed [18], direct evidence tracing the continuous duplication and functional refinement of a single gene lineage, as documented here, remains scarce.

Comparative genomic analysis across 48 plant species showed that *SDC* genes are broadly conserved, being present in 40 species—a finding consistent with their ancient evolutionary origin. *ASDC2* was detected within the order Ericales, including the family Theaceae, whereas *ASDC1* and *AlaDC* appear to be restricted to Theaceae (Fig. 3A). These distribution patterns suggest an evolutionary trajectory of *SDC*→*ASDC1* → *ASDC2* → *AlaDC*. Transcriptomic data further revealed that *SDC* is expressed across all 19 examined *Camellia* species, *ASDC2* in 17 species, while *ASDC1* and *AlaDC* transcripts were limited to a few species such as *C. sinensis* and *C. oleifera*. This restricted expression is characteristic of evolutionarily younger genes [28](Fig. 2B-C). Notably, *CsASDC2* was expressed at higher levels than *CsASDC1*, aligning with its broader phylogenetic distribution across *Camellia* species (Fig. 1E, F).

### Identification of key residues in the functional evolution from CsSDC to CsAlaDC

To decipher the molecular basis of the functional shift from CsSDC to CsAlaDC, we employed an integrated approach that combined comparative sequence analysis, structural modeling, and *in vitro* and *in planta* functional assays. An alignment of homologous sequences identified 21 amino acid positions that are strictly conserved among SDC proteins but are consistently substituted in CsAlaDC (Fig. S4). Structurally, decarboxylases generally consist of an N-terminal domain, a C-terminal domain, and a large core catalytic domain that governs substrate specificity [29, 30]. This architecture supports functional plasticity, as illustrated by Komori *et al* [20], who showed that a single substitution (S354G) in human histidine decarboxylase switches its substrate preference to dopa. In line with this precedent, our structural modeling placed the 21 divergent residues predominantly within the catalytic core of CsAlaDC/SDC. Strikingly, reciprocally swapping these 21 residues between CsAlaDC and CsSDC resulted in a complete functional exchange (Fig. 4B), providing definitive evidence that these sites constitute the key molecular determinants responsible for their functional divergence.

Through further functional screening, we narrowed the 21 candidate sites to five critical residues (Fig. 4C, D). Two substitutions emerged as particularly pivotal for the functional evolution from CsSDC to CsAlaDC: TAC (Tyr) → TTT (Phe) at position 106 and TGT (Cys) → GGT (Gly) at position 168 (Fig. 4E). Structural modeling indicates that Phe^106^ and Gly^168^ are located within a flexible loop near the catalytic center, and both were found to be essential for CsAlaDC activity. This is consistent with the established role of flexible loops in modulating catalytic dynamics and substrate recognition in decarboxylases [20, 31, 32]. The functional impact of single amino acid substitutions is well documented, exemplified by the conversion of aromatic amino acid decarboxylases to aromatic acetaldehyde synthases through a single Y350F mutation [33]. Our results align with and extend the earlier structural work of Wang *et al.* [21], who also identified Phe106 as a critical residue for CsAlaDC substrate specificity. Their observation that interface mutations (L110F and P114A) increased catalytic activity by 110% and 59%, respectively, further highlights the potential of structure-guided engineering to enhance the catalytic performance of AlaDC enzymes.

A key observation was that none of the single-site mutations in CsAlaDC conferred serine decarboxylase activity, indicating that the native function of CsSDC likely depends on cooperative interactions among multiple residues. This finding is similar to reports for other decarboxylases, such as human glutamic acid decarboxylase, where a shift in substrate specificity required the substitution of three conserved residues within the substrate-binding pocket [34]. Interestingly, single mutations in CsSDC frequently resulted in a total loss of activity, whereas most CsAlaDC mutants retained partial function (Fig. 4E). This disparity suggests that CsAlaDC possesses greater intrinsic structural plasticity than CsSDC. This flexibility may be rooted in their distinct metabolic roles: CsSDC operates within a conserved primary metabolic pathway, which typically imposes stricter structural constraints, while CsAlaDC functions in the specialized and more recently evolved theanine biosynthesis pathway, where structural adaptability could be advantageous for catalytic innovation [35].

## Conclusion

**Figure 6:**
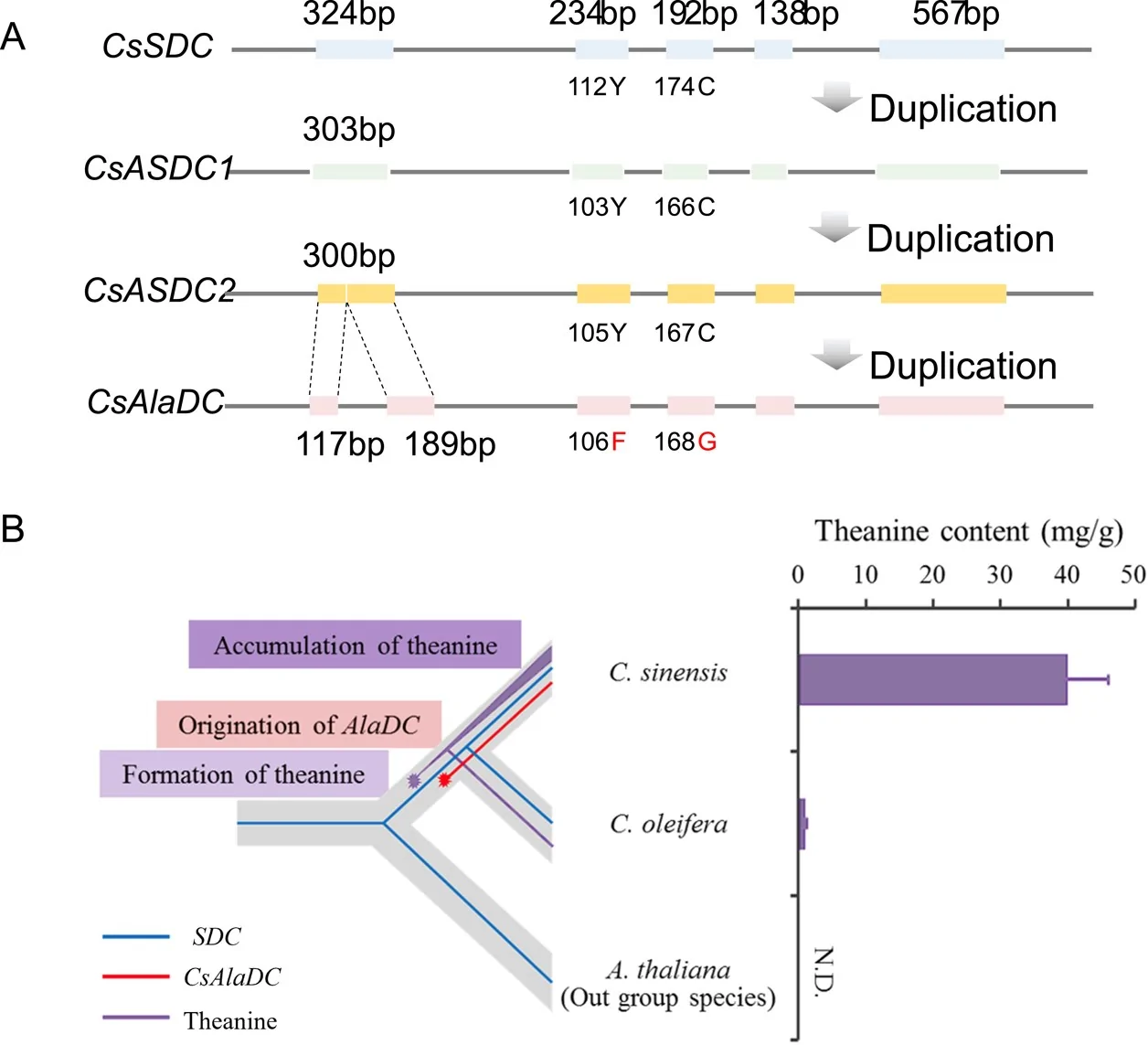
Evolutionary origin and functional role of *CsAlaDC* in theanine biosynthesis in *C. sinensis*. (A) Proposed evolutionary trajectory of *CsAlaDC* via gene duplication and subsequent neofunctionalization from the ancestral *CsSDC*. Gene structures of *CsSDC*, *CsASDC1*, *CsASDC2*, and *CsAlaDC* are depicted, with exon lengths indicated in base pairs (bp). Key residues for enzymatic function are highlighted. During evolution, codon substitutions TAC(Tyr) → TTT(Phe) and TGT(Cys) → GGT(Gly) gave rise to Phe^106^ and Gly^168^ in CsAlaDC, both located near the catalytic center and essential for alanine decarboxylase activity. Notably, the combined length of the first two exons of *CsAlaDC* approximates that of the first exon of *CsSDC*, suggesting an exon-splitting event post-duplication. (B) Model illustrating the contribution of CsAlaDC to theanine biosynthesis. The emergence of CsAlaDC enables efficient ethylamine production, promoting theanine synthesis and accumulation in *C. sinensis*. Theanine content in C. sinensis, *C. oleifera*, and the outgroup *A. thaliana* is shown for comparison.

In summary, a genome-wide survey in tea plants identified the key enzyme CsAlaDC alongside its homologs, including the ancestral serine decarboxylase CsSDC and two evolutionarily intermediate genes, CsASDC1 and CsASDC2. Phylogenetic reconstruction supports a model wherein the tea-specific *CsAlaDC* originated via gene duplication and subsequent neofunctionalization of a conserved *SDC* ancestor. Through integrated sequence, structural, and functional analyses—including site-directed mutagenesis and *in planta* validation—we demonstrated that two residues proximal to the catalytic center, Phe^106^ and Gly^168^, are indispensable for CsAlaDC activity. Critically, the corresponding codon substitutions TAC(Tyr112) → TTT(Phe106) and TGT(Cys174) → GGT(Gly168) emerged as pivotal molecular events driving the functional shift from CsSDC to CsAlaDC (Fig. 6A). Together, these findings delineate the evolutionary trajectory that gave rise to the neofunctionalized CsAlaDC, a central catalyst in the biosynthesis of theanine in tea plants (Fig. 6B).

## Materials and methods

### Experimental materials

This study utilized one-year-old seedlings of three tea cultivars: *Camellia sinensis* ‘Longjing 43’ (LJ43), *C. sinensis* ‘Zhongcha 108’ (ZC108), and *C. sinensis* ‘Zhongcha 302’ (ZC302). Seedlings of *Camellia oleifera* and *Camellia sasanqua* were provided by the Experimental Forest Farm of the Subtropical Forestry Research Institute, Chinese Academy of Forestry. *Nicotiana benthamiana*, *Arabidopsis thaliana*, and *Solanum lycopersicum* plants were maintained under standard greenhouse conditions in our laboratory. All plants were grown in a greenhouse until sampling. Collected tissues were immediately frozen in liquid nitrogen, stored at −80°C, and subsequently freeze-dried and ground into a fine powder for further analysis.

### Gene cloning, bioinformatics analysis, and RT-qPCR

Full-length coding sequences of *CsAlaDC* and *CsSDC* were amplified from *C. sinensis* ‘Longjing 43’ (LJ43). Homology searches via BLAST against the reference tea genome (*C. sinensis* ‘Shuchazao’) identified two additional homologs, designated *CsASDC1* and *CsASDC2*. Bioinformatics analyses, including primer design and sequence characterization, were conducted as previously described [36], all the software and tools used for bioinformatics analysis are listed in Table S1. For RT-qPCR, reactions were prepared using SYBR Green dye and run on a Roche LightCycler 480 II Real-Time PCR system. *CsGAPDH and NbACTIN* were used as the internal reference gene for normalization. All primer sequences are listed in Table S2. Relative expression levels were calculated using the 2^−ΔΔCt^ method and presented as mean ± SD from three biological replicates.

### Subcellular localization of CsAlaDC

For subcellular localization analysis, the full-length coding sequence of *CsAlaDC* was amplified from *C. sinensis* ‘Longjing 43’ and cloned into a GFP fusion vector under the control of the CaMV 35S promoter. The resulting construct (35S: *CsAlaDC*-GFP) and an EV control (35S: GFP) were individually introduced into *Agrobacterium tumefaciens* strain GV3101. Positive transformants were selected on solid medium supplemented with kanamycin. Bacterial cultures were harvested, resuspended in infiltration buffer (10 mM MES, 10 mM MgCl₂, 0.2 mM acetosyringone, pH 5.7), and infiltrated into the abaxial surface of young *N. benthamiana* leaves. After 48 hours of incubation under low-light conditions, GFP fluorescence was observed and imaged using a confocal laser scanning microscope (Nikon C2-ER, Japan).

### Stable genetic transformation of *A. thaliana*

*Agrobacterium thaliana* was stably transformed using the floral dip method. *Agrobacterium tumefaciens* strain GV3101, carrying the binary vector pBWA(V)BS-CsAlaDC, was grown in LB medium with kanamycin (50 μg/ml) to an OD_600_ of 1.0. The bacterial cells were pelleted and resuspended in Murashige and Skoog (MS) infusion buffer (5% sucrose, 0.02% Silwet L-77, pH 5.7). Flowering inflorescences were dipped into the suspension for 2–3 seconds, then kept under high humidity (>90%) in darkness at 25°C for 24 hours. The dipping procedure was repeated once after a 7-day interval.

After transformation, plants were grown under long-day conditions until seed set. Harvested T₀ seeds were dried and stored at 4°C. For selection, T₀ seeds were sown directly on soil, and emerging T₁ seedlings were sprayed with Basta (15 mg/l). Resistant plants were validated by PCR amplification of the *CsAlaDC* transgene. T₂ seeds from positive T₁ lines were surface-sterilized, stratified at 4°C for 2–3 days, and germinated on half-strength MS medium containing Basta. The same selection process was applied to obtain T₃ seeds. Lines showing 100% survival on selective medium were considered homozygous and used for subsequent experiments.

### Measurement of fresh weight and primary root length in *A. thaliana* seedlings

Seeds from two homozygous transgenic lines and the wild type (WT) were surface-sterilized and sown on square agar plates. After stratification at 4°C in darkness for 3 days, plates were transferred to a growth chamber under standard long-day conditions (16-hour light/8-hour dark, 22°C).

Following two weeks of growth, the fresh weight and primary root length of individual seedlings were measured. For each genotype, 24 seedlings were assessed as biological replicates. Statistical significance was determined using one-way analysis of variance (ANOVA) in SPSS software.

### Determination of theanine and ethylamine content

The contents of theanine and ethylamine were determined based on a previously established method [37]. Briefly, tissue samples were ground to a fine powder in liquid nitrogen and then lyophilized at −55°C for 3 days until constant weight was achieved. Metabolites were extracted following the Chinese National Standard protocol GB/T 23193-2008 and quantified by ultra-performance liquid chromatography (UPLC, Waters, USA).

### Phylogenetic inference and multiple sequence alignment

Phylogenetic analysis was inferred using the maximum likelihood method in MEGA X software. Branch support was assessed with 1000 bootstrap replicates. Multiple sequence alignments were generated using DNAMAN. The resulting phylogenetic tree was visualized and annotated with iTOL (https://itol.embl.de/).

### Chromosomal localization, and expression profiling of *AlaDC/SDC* genes in *Camellia* species

Chromosomal localization was visualized using TB tools and Map Chart software. Transcriptomic data for tea plant and 18 related *Camellia* species were obtained from the TPIA (https://tpia.teaplants.cn/), Table S3 for species list. Corresponding protein sequences were retrieved by local BLAST using thresholds of ≥50% coverage and ≥40% identity. Multiple sequence alignment and phylogenetic reconstruction were performed in MEGA X using the Neighbor-Joining method, and the resulting tree was visualized with iTOL. Tissue-specific expression data for *AlaDC/SDC* genes across eight tissues were extracted from the TPIA database, and expression patterns were visualized as heatmaps using TBtools [38].

### Phylogenetic analysis and genome-wide identification of *AlaDC/SDC* genes

Genome and protein sequence data for 48 plant species were retrieved from the plaBiPD database (https://www.plabipd.de) and published genomes [39, 40], species details in Table S4. Based on NCBI taxonomy IDs, a species tree was generated using LifeMap (http://lifemap-ncbi.univ-lyon1.fr/) and visualized in iTOL. To identify AlaDC/SDC homologs, BLASTP searches were performed with thresholds of ≥70% coverage and ≥50% identity. The copy number of identified genes was subsequently annotated onto the resulting phylogenetic tree.

### Phylogenetic analysis of AlaDC/SDC proteins

Protein sequences of AlaDC/SDC homologs from the 48 species were aligned using ClustalW in MEGA X. A phylogenetic tree was then constructed with the Neighbor-Joining method (1000 bootstrap replicates) to evaluate branch support. The resulting tree was visualized and annotated alongside the corresponding gene structures using iTOL. Conserved protein motifs were identified with the MEME suite (https://meme-suite.org/meme/).

### Identification of homologous protein and comparison of divergent residues

Protein homologs of CsSDC were identified by performing a BLASTP search against the NCBI RefSeq_protein database, retaining the top 100 non-redundant, full-length hits. These were supplemented with verified SDC sequences from *A. thaliana* and *Brassica napus* (UniProt). Following redundancy removal, a multiple sequence alignment was generated using ClustalW in MEGA-X to pinpoint residues that are conserved among SDC homologs but are substituted in CsAlaDC. Finally, the predicted 3D structures of CsSDC and CsAlaDC were compared and visualized in Swiss-PDBViewer to assess differences in their functional domain architecture.

### Site-directed mutagenesis, recombinant protein expression, purification, and enzyme activity assays

Site-directed mutations were introduced into the target gene via direct gene synthesis. The mutated coding sequences were then cloned into the pET-32a (+) vector via restriction digestion and ligation. Recombinant protein expression and purification followed a previously established protocol [15]. Briefly, *Escherichia coli* BL21(DE3) cells harboring the expression constructs for N-terminally His_6_-tagged proteins were induced with IPTG. Cells were harvested, lysed by ultrasonication, and the recombinant proteins were purified by Ni^2+^-NTA affinity chromatography. Protein purity was verified by SDS-PAGE and western blot analysis, and concentration was determined using the Bradford assay. Enzyme activity assays were performed in triplicate. Purified proteins were incubated with their respective substrates under optimized reaction conditions. Decarboxylase activity was determined by quantifying the reaction products via UPLC.

### Transient agroinfiltration in *N. benthamiana*

For transient overexpression, the full-length coding sequences of *CsAlaDC*, *CsSDC*, and their site-directed mutants were individually inserted into the plant expression vector pCAMBIA1302 under the control of the CaMV 35S promoter. Each construct was introduced into *Agrobacterium tumefaciens* strain GV3101(pSoup). Bacterial cultivation, harvest, resuspension, and leaf infiltration were performed as described for the subcellular localization assay. Each construct was infiltrated into leaves of at least three independent biological replicates. Leaf tissues were collected 60 hours post-infiltration, flash-frozen in liquid nitrogen, and stored at −80°C. These samples were subsequently used for ethylamine quantification and RT-qPCR analysis.

### Statistical analysis

Data are presented as mean ± standard deviation (SD) from at least three independent replicates. Significant differences were determined by one-way ANOVA, with pairwise comparisons conducted using Student’s *t*-tests where appropriate.

## Supplementary Material

Web_Material_uhag153

## Data Availability

All data, tables, and figures in this manuscript are original, and are contained within the article and supplementary materials.
