# Supplementary material for: Molecular evolution and functional divergence of CsAlaDC from CsSDC reveal key determinants for theanine biosynthesis in tea plants
Source: Hortic Res. 2026 Apr 17;13(9):uhag153. doi: 10.1093/hr/uhag153 (PMC13522723; doi:10.1093/hr/uhag153)
Supplement: Supplementary file 1 [file web_material_uhag153.zip › Supplementary_material_uhag153.docx]

Fig.S1 Subcellular localization of CsAlaDC in tobacco leaf.

Fig.S2 Multiple sequence alignment of CsSDC, CsASDC1, CsASDC2, and CsAlaDC.

Note: The conserved pyridoxal phosphate (PLP)-dependent decarboxylase domain is indicated by a red line. The conserved substrate-binding and catalytic residues are denoted by blue and green triangles, respectively.

Fig.S3 Predicted three-dimensional of CsAlaDC, CsSDC, CsASDC1 and CsASDC2 generated using the SWISS-MODEL homology modeling server.

Fig. S4 Distribution of variant residues between CsAlaDC and SDCs protein.

Note: The secondary structures of CsAlaDC and CsSDC proteins were predicted that are conserved among SDCs sequences across multiple species but divergent in the AlaDC sequence of tea plants.

| Function | Software/ Tool | Website |
| --- | --- | --- |
| Primer design | Primer-BLAST | <https://www.ncbi.nlm.nih.gov/tools/primer-blast/> |
| Pairwise sequence alignment | Needle | <https://www.ebi.ac.uk/Tools/psa/emboss_needle/> |
| Open reading frame (ORF) identification and translation | ORF fonder | <https://www.ncbi.nlm.nih.gov/orffinder/> |
| Prediction of physicochemical properties | ProtParam | http: //web.expasy.org/protparam/ |
| Homologous sequence retrieval | BLAST | <https://blast.ncbi.nlm.nih.gov/Blast.cgi> |
| Phylogenetic tree construction | MEGA-X | <https://www.megasoftware.net/> |
| Conserved domain prediction | CDD | <https://www.ncbi.nlm.nih.gov/cdd/> |
| Homology modeling | SWISS-MODEL | <https://swissmodel.expasy.org/> |
| Signal peptide prediction | SignalP | <http://www.cbs.dtu.dk/services/SignalP/> |
| Subcellular localization prediction | WoLFPSORT | <http://psort.hgc.jp/> |

Table S1 Software and tools used for bioinformatics analysis

Table S2 Primers used for gene cloning and qPCR

| Primer name | Sequence |
| --- | --- |
| *CsAlaDC -F* | 5′–CTCCTCGGATTCTCAAACCTCAC–3′ |
| *CsAlaDC -R* | 5′–CACACAAACGATAGTAGAACCAACA–3′ |
| *CsSDC-F* | 5’-TCTGACTTTTCCTCAAGCTCTG-3’ |
| *CsSDC-R* | 5’-CAGACAAAACAACAGAGGCC-3’ |
| *CsASDC1-F* | 5’-TTTTCCGTCATTATATCC-3’ |
| *CsASDC1-R* | 5’-ACAAATGACAGCAGAATC-3’ |
| *CsASDC2-F* | 5’-GGTAGGGACAGTAGAAGC-3’ |
| *CsASDC2-R* | 5’-GTAGAACCAACAAATAGC-3’ |
| *CsGAPDC-Q-F* | 5’-TTGGCATCGTTGAGGGTCT-3’ |
| *CsGAPDC-Q-R* | 5’-CAGTGGGAACACGGAAAGC-3’ |
| *CsSDC-Q-F* | 5’-ACCGGTGGTGATTGATTGCT-3’ |
| *CsSDC-Q-R* | 5’-GCTCGGTGACATCAAAGCAC-3’ |
| *CsASDC1-Q-F* | 5’-TAGACCTCCTTGTCTTGCAGC-3’ |
| *CsASDC1-Q-R* | 5’-TTAGCCATGAGTAGACGGGC-3’ |
| *CsASDC2-Q-F* | 5’-TGAGATCGGCAATGGAGG-3’ |
| *CsASDC2-Q-R* | 5’-GAAAGCCTCCTTATCGCC-3’ |
| *CsAlaDC-Q-F* | 5’-GAGTTGCTGGCGGAGAGAC-3’ |
| *CsAlaDC-Q-R* | 5’-GTCATCGTCCACATCAGGCT-3’ |
| *NbACTIN-F* | 5’-AGTCCTCTTCCAGCCATCCA-3’ |
| *NbACTIN-R* | 5’-TAGGAGCCAAAGCCGTGATT-3’ |

Table S3 Genome data used in this study

| Classification | Species name | Common name | Source of genomes | Genome accession/Project ID | NCBI Taxid ID |
| --- | --- | --- | --- | --- | --- |
| Bryophytes | *Anthoceros* *agrestis* | Hornworts | <https://doi.org/10.6084/m9.figshare.9974999> |  | 41834 |
|  | *Marchantia polymorpha* | Liverworts | NCBI | GCA_009936355.2 | 1480154 |
|  | *Ceratodon purpureus* | Pincushion moss | NCBI | GCA_014871385. 1 | 3225 |
|  | *Pleurozium schreberi* | Red-stemmed feathermoss | [https://github.com/PycnopodiaD/](https://github.com/PycnopodiaD/Pleurozium_schreberi_genome_files)Pleurozium_schreberi_genome_files |  | 34163 |
| Ferns | *Selaginella tamariscina* | Spikemosses | NCBI | GCA_003024785. 1 | 137178 |
|  | *Isoetes engelmannii* | Quillworts | NCBI | GCA_011763485. 1 | 37427 |
| Gymnosperms | *Ginkgo biloba* | Ginkgo | GigaDB | PRJNA307642 | 3311 |
|  | *Gnetum montanum* | Jointfirs | NCBI | GCA_015680685. 1 | 3381 |
| Angiosperms | *Musa balbisiana* | Wild banana | NCBI | GCA_004837865. 1 | 52838 |
|  | *Oryza sativa* | Rice | NCBI | GCA_014636035. 1 | 4530 |
|  | *Zea mays* | Maize | NCBI | GCA_902714155. 1 | 4577 |
|  | *Dendrobium officinale* | Dendrobium | GenBank | SUB764497 | 142615 |
|  | *Dioscorea Cayenensis* | Chinese yam | NCBI | GCA_009730915. 1 | 55577 |

Table S3 Continued from genome data used in this study

| Classification | Species name | Common name | Source of genomes | Genome accession/Project ID | NCBI Taxid ID |
| --- | --- | --- | --- | --- | --- |
| Angiosperms | *Chimonanthus praecox* | Wintersweet | NCBI | PRJNA600650 | 13419 |
|  | *Nymphaea colorata* | Water lily | NCBI | GCA_902499525. 1 | 210225 |
|  | *Papaver somniferum* | Poppy | NCBI | GCA_010119995. 1 | 3469 |
|  | *Actinidia eriantha* | Kiwifruit | GigaDB | PRJNA480681 | 165200 |
|  | *Citrus medica* | Citron | NCBI | GCA_002013955.2 | 171251 |
|  | *Arabidopsis thaliana* | Thale cress | NCBI | GCA_903064285. 1 | 3702 |
|  | *Brassica napus* | Rapeseed | NCBI | GCA_000686985.2 | 3708 |
|  | *Gossypium hirsutum* | Upland cotton | NCBI | GCA_006980745. 1 | 3635 |
|  | *Punica granatum* | Pomegranate | NCBI | GCA_007655135.2 | 22663 |
|  | *Populus alba* | Poplar | NCBI | GCA_005239225. 1 | 43335 |
|  | *Cucumis sativus* | Cucumber | NCBI | GCA_000004075.3 | 3659 |
|  | *Juglans regia* | Walnut | NCBI | GCA_001411555.2 | 51240 |
|  | *Prunus persica* | Peach | NCBI | GCA_015730445. 1 | 3760 |

Table S3 Continued from genome data used in this study

| Classification | Species name | Common name | Source of genomes | Genome accession/Project ID | NCBI Taxid ID |
| --- | --- | --- | --- | --- | --- |
| Angiosperms | *Glycine max* | Soybean | NCBI | GCA_003349995.2 | 3847 |
|  | *Chrysanthemum seticuspe* | Roman chamomile | NCBI | GCA_004359105. 1 | 1111766 |
|  | *Solanum lycopersicum* | Tomato | NCBI | GCA_012431665.1 | 4081 |
|  | *Nicotiana tabacum* | Tobacco | NCBI | GCA_000715135.1 | 4097 |
|  | *Olea europaea* | Olive | NCBI | GCA_902713445.1 | 158383 |
|  | *Coffea arabica* | Coffee | NCBI | GCA_003713225.1 | 13443 |
|  | *Hydrangea macrophylla* | Hydrangea | NCBI | GCA_013391905.1 | 23110 |
|  | *Amaranthus hypochondriacus* | Grain amaranth | NCBI | GCA_000753965.2 | 28502 |
|  | *Chenopodium quinoa* | Quinoa | NCBI | GCA_001683475.1 | 63459 |
|  | *Rhododendron simsii* | Rhododendron | NCBI | GCA_014282245.1 | 118357 |
|  | *Vaccinium corymbosum* | Highbush blueberry | NCBI | GCA_014504835.1 | 69266 |
|  | *Primula veris* | Cowslip | NCBI | GCA_000788445.1 | 170927 |
|  | *Diospyros oleifera* | Oil persimmon | NCBI | PRJNA532832 | 227308 |
|  | *Clethra arborea* | Cherry | <https://doi.org/10.5061/dryad.573n5tb4k> |  | 59776 |

Table S3 Continued from genome data used in this study

| Classification | Species name | Common name | Source of genomes | Genome accession/Project ID | NCBI Taxid ID |
| --- | --- | --- | --- | --- | --- |

|  | *Fragaria nilgerrensis* | | | Strawberry | | NCBI | | GCA_010134655. 1 | | 64941 | |
| --- | --- | --- | --- | --- | --- | --- | --- | --- | --- | --- | --- |
|  | | *Spirodela polyrhiza* | Common duckweed | | NCBI | | GCA_013350415. 1 | | 29656 | |  |

| Angiosperms | *Roridula gorgonias* | Cape bug plant | [https://doi.org/10.5061/dryad.573n](https://doi.org/10.5061/dryad.573n5tb4k)5tb4k |  | 3772 |
| --- | --- | --- | --- | --- | --- |
|  | *Synsepalum dulcificum* | Miracle fruit | CNGBdb | CNP0002330 | 3743 |
|  | *Camellia chekiangoleosa* | Red-flowered oil-tea | NGDC | GWHBGBN00000000 | 450940 |
|  | *Camellia oleifera* | Oil-tea camellia | <https://github.com/Hengfu-Yin/CON_genome_data> |  | 385388 |
|  | *Camellia sinensis* | Tea plant | NCBI | GCA_013676235. 1 | 4442 |

Table S4 Taxonomic information and species names of 19 species of Camellia plants

| Genus | Subgenus | Section | Series | Species name | Common name |
| --- | --- | --- | --- | --- | --- |
| Camellia | Camellia | Thea | Sinenses | *Camellia* *angustifolia* | Narrow-leaf Tea |
| Camellia | Camellia | Thea | Sinenses | *Camellia* *parvisepala* | Small-sepal Tea |
| Camellia | Camellia | Thea | Sinenses | *Camellia* *ptilophylla* | Rucheng Hairy-leaf Tea |
| Camellia | Camellia | Thea | Sinenses | *Camellia* *pubescens* | Hairy-leaf Tea |
| Camellia | Camellia | Thea | Sinenses | *Camellia* *sinensis* | Tea |
| Camellia | Camellia | Thea | Gymnogynae | *Camellia* *gymnogyna* | Bare-ovary Tea |
| Camellia | Camellia | Thea | Gymnogynae | *Camelliajingyunshanica* | Jingyun Mountain Tea |
| Camellia | Camellia | Thea | Gymnogynae | *Camellia* *leptophylla* | Thin-leaf Tea |
| Camellia | Camellia | Thea | Pentastylae | *Camellia* *kwangsiensis* | Guangxi Tea |
| Camellia | Camellia | Thea | Pentastylae | *Camellia* *tachangensis* | Dachang Tea |
| Camellia | Camellia | Thea | Pentastylae | *Camellia* *tetracocca* | Dachang Tea |
| Camellia | Camellia | Thea | Pentastylae | *Camellia* *atrothea* | Laohei Tea |
| Camellia | Camellia | Thea | Pentastylae | *Camellia* *crassicolumna* | Thick-column Tea |

| Genus | Subgenus | Section | Series | Species name | Common name |
| --- | --- | --- | --- | --- | --- |
| Camellia | Camellia | Thea | Pentastylae | *Camellia* *makuanica* | Maguan Tea |
| Camellia | Camellia | Thea | Pentastylae | *Camellia* *taliensis* | Dali Tea |
| Camellia | Camellia | Camellia | Reticulatae | *Camellia* *reticulata* | Yunnan Camellia |
| Camellia | Camellia | Camellia | Reticulatae | *Camellia* *chekiangoleosa* | Red-flower Oil-tea Camellia |
| Camellia | Camellia | Oleifera | Oleiferae | *Camellia* *oleifera* | Tea-oil Camellia |
| Camellia | Camellia | Oleifera | Sasanquae | *Camellia* *sasanqua* | Sasanqua |

Table S4 Continued from taxonomic information and species names of 19 species of Camellia plants

Table S5 Distribution of theanine and ethylamine in different species

| Species/ Varieties | Theanine content (mg/g) | | Ethylamine content (mg/g) | |
| --- | --- | --- | --- | --- |
|  | root | leaf | root | leaf |
| *Camellia* *sinensis* cv. zhongcha 108 | 20.094 ± 1.964 | 0.981 ± 0.059 | 0.195 ± 0.009 | 0.037 ± 0.004 |
| *Camellia* *sinensis* cv. zhongcha 302 | 15.781 ± 0.395 | 1.642 ± 0.020 | 0.121 ± 0.003 | 0.038 ± 0.001 |
| *Camellia* *sinensis* cv. longjing 43 | 14.803 ± 0.217 | 7.210 ± 0.448 | 0.182 ± 0.004 | 0.037 ± 0.001 |
| *Camellia* *oleifera* | 2.814 ± 0.054 | 0.243 ± 0.039 | 0.049 ± 0.001 | 0.018 ± 0.000 |
| *Camellia* *sasanqua* | 1.001 ± 0.012 | N.D. | 0.037 ± 0.001 | N.D. |
| *Arabidopsis* *thaliana* | N.D. | N.D. | N.D. | N.D. |
| *Solanum* *lycopersicum* | N.D. | N.D. | N.D. | N.D. |

Table S6 Predicted physical and chemical parameters of CsAlaDC/SDC

| Parameters | CsSDC | CsASDC1 | CsASDC2 | CsAlaDC |
| --- | --- | --- | --- | --- |
| Number of amino acid residues | 484 | 476 | 477 | 478 |
| Molecular weight (kDa) | 54.7 | 53.4 | 53.5 | 53.6 |
| Theoretical isoelectric point (pI) | 5.71 | 6.13 | 5.92 | 5.83 |
| Protein stability index | Stable | Inherently unstable | Inherently unstable | Inherently unstable |
| Instability index | (38.07) | (43.02) | (42.40) | (37.04) |
| Hydrophobicity profile | Hydrophilic | Hydrophilic | Hydrophilic | Hydrophilic |
| Grand average of hydropathy | (-0.243) | (-0.210) | (-0.180) | (-0.205) |
| Signal peptide prediction | Not detected | Not detected | Not detected | Not detected |
| Subcellular localization prediction | Cytoplasmic | Cytoplasmic | Cytoplasmic | Cytoplasmic |

Table S7 Variant sites of CsAlaDC with SDCs from tea plants and other species

Note：The amino acid residues marked in red font represent three positions located at the enzyme's catalytic center, while the amino acid residues marked in blue font represent two positions outside the enzyme's catalytic center.

| Number / No. | | Site | | SDCs | CsAlaDC |
| --- | --- | --- | --- | --- | --- |
| 1 | | 83 | | G | S |
| 2 | | 112 | | Y | F |
| 3 | | 116 | | F | L |
| 4 | | 120 | | A | P |
| 5 | | 123 | | Q | E |
| 6 | | 128 | | S | A |
| 7 | | 174 | | C | G |
| 8 | | 195 | | L | F |
| 9 | | 197 | | A | T |
| 10 | | 248 | | V | L |
| 11 | | 261 | | L | I |
| 12 | | 289 | | L | F |
| 13 | | 294 | | V | L |
| 14 | | 295 | | K | N |
| 15 | | 297 | | A | G |
| 16 | | 317 | | V | M |
| 17 | | 321 | | M | T |
| 18 | 351 | | M | | T |
| 19 | 358 | | A | | S |

Table S7 Continued from Variant sites of CsAlaDC with SDCs from tea plants and other species.

| Number / No. | Site | SDCs | CsAlaDC |
| --- | --- | --- | --- |
| 20 | 361 | F | I |
| 21 | 371 | Y | F |
